# Microbe-derived uremic solutes enhance thrombosis potential in the host

**DOI:** 10.1128/mbio.01331-23

**Published:** 2023-11-10

**Authors:** Ina Nemet, Masanori Funabashi, Xinmin S. Li, Mohammed Dwidar, Naseer Sangwan, Sarah M. Skye, Kymberleigh A. Romano, Tomas Cajka, Brittany D. Needham, Sarkis K. Mazmanian, Adeline M. Hajjar, Federico E. Rey, Oliver Fiehn, W. H. Wilson Tang, Michael A. Fischbach, Stanley L. Hazen

**Affiliations:** 1 Department of Cardiovascular & Metabolic Sciences, Lerner Research Institute, Cleveland, Ohio, USA; 2 Center for Microbiome & Human Health, Cleveland Clinic, Cleveland, Ohio, USA; 3 Department of Bioengineering, Stanford University, Stanford, California, USA; 4 Department of Microbiology and Immunology, Stanford University School of Medicine, Stanford University, Stanford, California, USA; 5 ChEM-H Institute, Stanford University, Stanford, California, USA; 6 West Coast Metabolomics Center, University of California, Davis, California, USA; 7 Departments of Biology and Biological Engineering, California Institute of Technology, Pasadena, California, USA; 8 Department of Bacteriology, University of Wisconsin-Madison, Madison, Wisconsin, USA; 9 Heart and Vascular Institute, Cleveland Clinic, Cleveland, Ohio, USA; 10 Chan Zuckerberg Biohub, San Francisco, California, USA; University of Hawaii at Manoa, Honolulu, Hawaii, USA; University of Pennsylvania, Philadelphia, Pennsylvania, USA

**Keywords:** gut microbes, uremic toxins, *p*-cresol sulfate, indoxyl sulfate, cardiovascular disease, mortality

## Abstract

*p*-Cresol sulfate (*p*CS) and indoxyl sulfate (IS), gut microbiome-derived metabolites, are traditionally associated with cardiovascular disease (CVD) risks in the setting of impaired kidney function. While pharmacologic provision of *p*CS or IS can promote pro-thrombotic phenotypes, neither the microbial enzymes involved nor direct gut microbial production have been linked to CVD. Untargeted metabolomics was performed on a discovery cohort (*n* = 1,149) with relatively preserved kidney function, followed by stable isotope-dilution mass spectrometry quantification of *p*CS and IS in an independent validation cohort (*n* = 3,954). Genetic engineering of human commensals to produce *p*-cresol and indole gain-of-function and loss-of-function mutants, followed by colonization of germ-free mice, and studies on host thrombosis were performed. Systemic *p*CS and IS levels were independently associated with all-cause mortality. Both *in vitro* and within colonized germ-free mice *p*-cresol productions were recapitulated by collaboration of two organisms: a *Bacteroides* strain that converts tyrosine to 4-hydroxyphenylacetate, and a *Clostridium* strain that decarboxylates 4-hydroxyphenylacetate to *p-*cresol. We then engineered a single organism, *Bacteroides thetaiotaomicron*, to produce *p-*cresol, indole, or both metabolites. Colonizing germ-free mice with engineered strains*,* we show the gut microbial genes for *p-*cresol (*hpdBCA*) and indole (*tryptophanase*) are sufficient to confer a pro-thrombotic phenotype *in vivo*. Moreover, human fecal metagenomics analyses show that abundances of *hpdBCA* and *tryptophanase* are associated with CVD. These studies show that *p*CS and IS, two abundant microbiome-derived metabolites, play a broader potential role in CVD than was previously known. They also suggest that therapeutic targeting of gut microbial *p-*cresol- and indole-producing pathways represent rational targets for CVD.

**IMPORTANCE:**

Alterations in gut microbial composition and function have been linked to numerous diseases. Identifying microbial pathways responsible for producing molecules that adversely impact the host is an important first step in the development of therapeutic interventions. Here, we first use large-scale clinical observations to link blood levels of defined microbial products to cardiovascular disease risks. Notably, the previously identified uremic toxins *p*-cresol sulfate and indoxyl sulfate were shown to predict 5-year mortality risks. After identifying the microbes and microbial enzymes involved in the generation of these uremic toxins, we used bioengineering technologies coupled with colonization of germ-free mice to show that the gut microbial genes that generate *p*-cresol and indole are sufficient to confer *p*-cresol sulfate and indoxyl sulfate formation, and a pro-thrombotic phenotype *in vivo*. The findings and tools developed serve as a critical step in both the study and targeting of these gut microbial pathways *in vivo*.

## INTRODUCTION

Cardiovascular disease (CVD) remains the leading cause of mortality worldwide; however, despite significant advances in preventive CVD treatments and prevention efforts, there remains a significant “residual CVD risk,” with numerous atherothrombotic events and cardiovascular mortality risk occurring even among optimally treated individuals (1–4). The search for additional causative factors beyond traditional CVD risk factors remains a significant area of investigation. One promising area in this regard is the gut microbiome (5, 6). Differences in gut microbial composition and function have been associated with CVD, including production of some bioactive metabolites (5, 7–10). While most of studies on gut microbes and CVD are correlative and associative, multiple mechanistic studies are identifying gut microbial metabolites as direct contributors to CVD (7, 11–16). *p*-Cresol sulfate (*p*CS) and indoxyl sulfate (IS), products derived from microbial fermentation of the aromatic amino acids tyrosine and tryptophan, respectively, are associated with CVD mortality risk in individuals with chronic kidney disease (CKD) and end-stage renal disease (17–19).

Termed “uremic solutes” because levels of *p*CS and IS accumulate as renal function declines, these compounds are also considered “uremic toxins” because they have been implicated in mediating adverse phenotypes in the setting of renal disease (20). The synthesis of both *p*CS and IS are also “metaorganismal,” since each is co-synthesized by an initial (obligate) gut microbiota-dependent process, followed by the host enzymatic transformations that facilitate metabolite excretion. Each molecule begins as a byproduct of bacterial fermentation of aromatic amino acids in a protein-rich diet. For IS production, tryptophan from the diet can be degraded into indole by microbial tryptophanase genes encoded in the genome of a variety of gut commensals, as we recently showed (21). The gut microbiota-derived indole, once absorbed via the portal circulation, can then be oxidized by CYP2E1 and sulfated by SULT1A1, both endogenous host enzymes. Synthesis of *p*CS follows a similar overall trajectory. Dietary protein-derived tyrosine is metabolized by gut microbes via various mechanisms to yield *p*-cresol, which, following absorption into the host, can then undergo sulfation by SULT1A1 to yield *p*CS (22, 23). While the host enzymes in these metaorganismal biochemical transformations are well-known and characterized, the gut microbes and the microbial genes utilized to produce the precursor molecules in these metaorganismal pathways remain poorly understood.

Our interest in *p*CS and IS in the present studies began as a result of untargeted metabolomics investigations, which identified microbial precursor of *p*CS as candidate molecule whose circulating (blood) levels predict incident CVD risks among subjects with normal renal function. Through subsequent targeted mass spectrometry analysis on an independent cohort, we now show that *p*CS and IS are associated with overall mortality in individuals with preserved kidney function. We then identify a missing link in the microbial biosynthetic pathway of *p*-cresol and take advantage of this finding to engineer strains of *Bacteroides thetaiotaomicron* that produce *p*-cresol, indole, or both. By colonizing germ-free mice with these strains, we confirm that microbial genes responsible for production of either *p*-cresol or indole lead to formation of *p*CS and IS, respectively. We then further demonstrate that this is sufficient to confer within the host, formation of either *p*CS or IS, and induction of a pro-thrombotic phenotype. Finally, in human metagenomics analyses, we show the fecal abundances of microbial *hpdBCA* and *tryptophanase* genes are independently associated with CVD. Our work, thus, demonstrates that microbiota targeting therapies aimed at reducing levels of these uremic solutes/toxins should be explored, and suggests potential microbial enzymatic transformations as rational therapeutic targets for treatment of residual CVD risk.

## RESULTS

### Uremic solutes are associated with overall mortality in individuals with preserved kidney function

**Fig 1:**
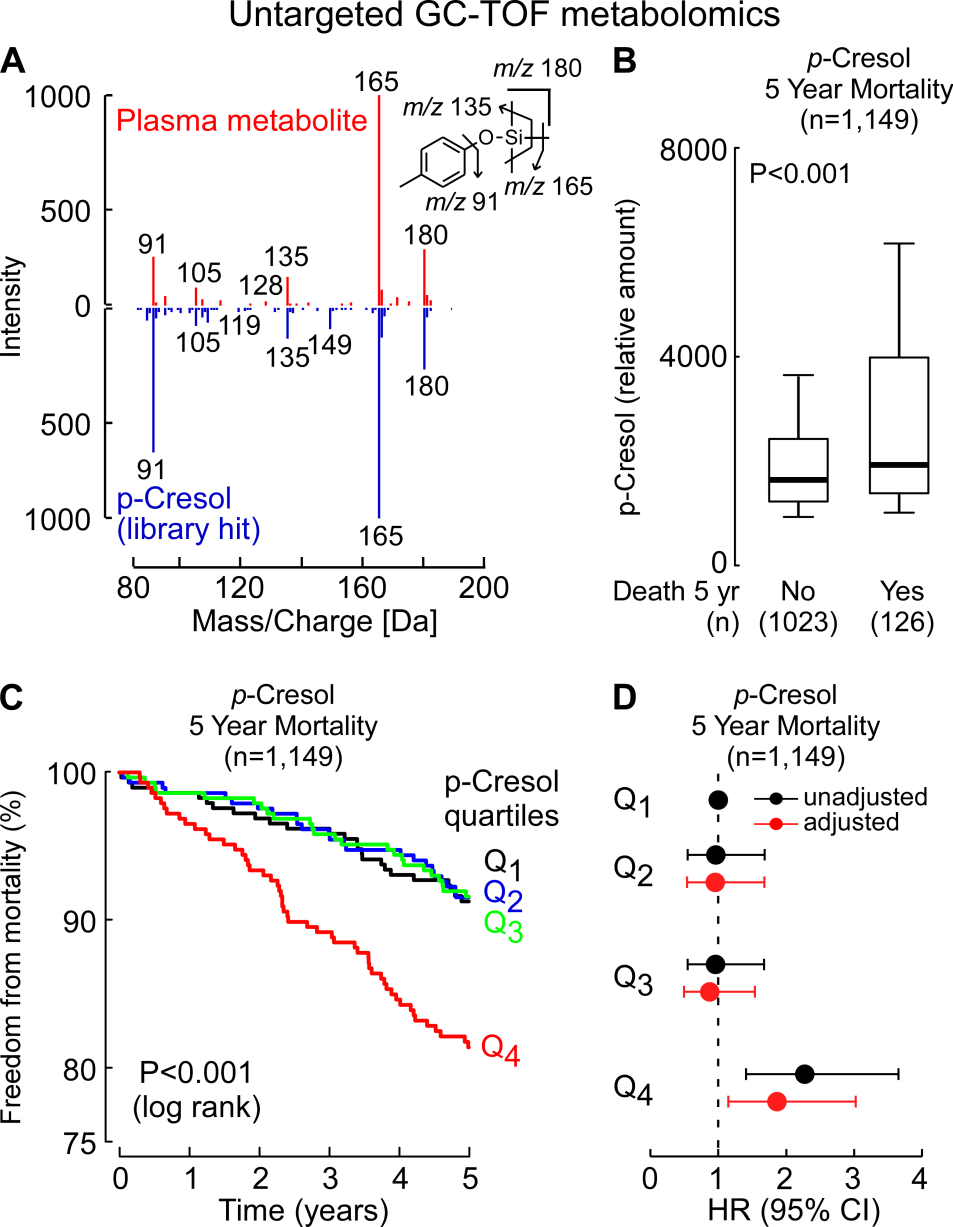
Untargeted metabolomics reveals that *p*-cresol is associated with overall mortality. (**A**) Comparison of electron ionization spectra of the compound detected in plasma and an authentic standard of the trimethylsilyl derivative of *p*-cresol. (**B**) Relative plasma levels of *p*-cresol in sequential stable subjects undergoing elective diagnostic cardiac evaluation. Subjects (*n* = 1,149; discovery cohort) were divided into groups as indicated based on whether or not they died during the 5-year follow-up. In the box-whisker plot, the upper and lower boundaries of the box represent the 25th and 75th percentiles, the median is marked by a horizontal line inside the box, and the whiskers represent 10% and 90% measured values. (**C**) Kaplan-Meier estimates of the risk of incident death by quartile of relative amounts of *p*-cresol from the untargeted analysis. (**D**) Forest plots showing overall mortality within 5 years among test subjects according to the quartiles for the relative level of *p*-cresol (black), or a multivariable Cox model for hazard ratio that includes adjustments for age, sex, current smoking, high-density lipoprotein, low-density lipoprotein, triglyceride level, systolic blood pressure, diabetes mellitus, and high-sensitivity C-reactive protein (adjusted, red). Symbols represent hazard ratios and the 95% CIs are indicated by the line length.

Plasma samples from individuals undergoing elective diagnostic cardiac evaluation with longitudinal follow-up (*n* = 1,149; Table S1) were analyzed using untargeted gas chromatography time-of-flight mass spectrometry (GC-MS-TOF) as described under Materials and Methods. A derivatized analyte consistent with *p*-cresol (Fig. 1A), the microbiome-derived metabolite and precursor to *p*CS, was noted to be significantly higher among subjects who died over the ensuing period of follow-up (5 years) compared to those that did not (Fig. 1B; *P* < 0.0001). Kaplan-Meier survival analyses also suggested that levels of the candidate ion identified as *p*-cresol were associated with incident mortality risk in this cohort (Fig. 1C). Subsequent Cox proportional hazards regression with time-to-event analysis showed subjects with elevated (fourth quartile [Q4]) levels had higher mortality risk compared to those with low (first quartile [Q1]) levels, even after adjustment for traditional risk factors (hazard ratio [HR] 95% confidence interval [CI] for incident [5 years] death risk of HR = 1.86 [1.15–3.03], *P* = 0.01) (Fig. 1D).

**Fig 2:**
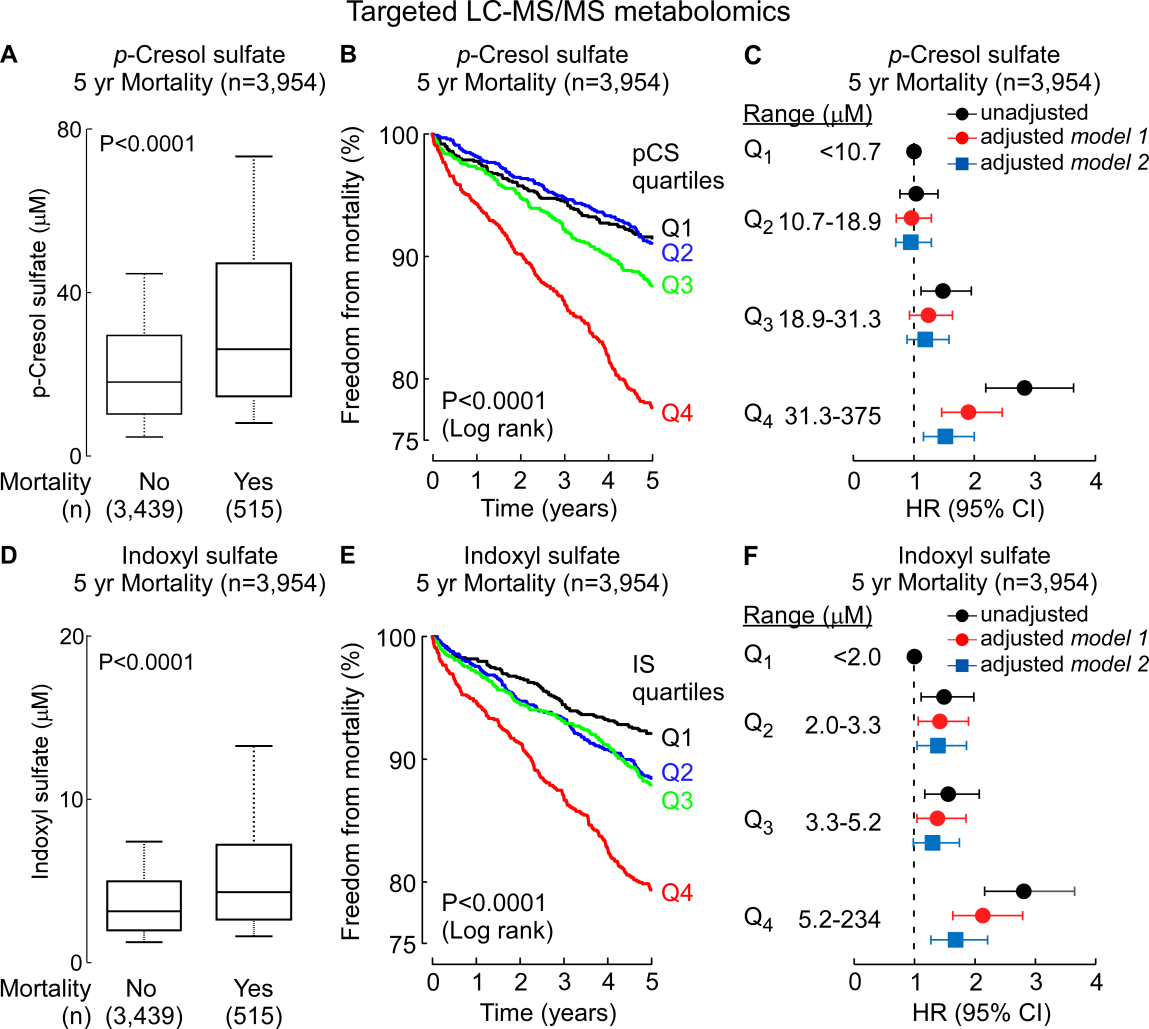
Stable isotope-dilution LC-MS/MS analyses verify systemic levels of uremic toxins *p*-cresol sulfate and indoxyl sulfate are associated with incident overall mortality risks. Plasma levels of *p*-cresol sulfate (**A**) and indoxyl sulfate (**D**) in sequential stable subjects undergoing elective diagnostic cardiac evaluation. Subjects (*n* = 3,954) were divided into groups as indicated based on whether or not they experienced an incident death event within 5 years. In the box-whisker plot, the upper and lower boundaries of the box represent the 25th and 75th percentiles, the median is marked by a horizontal line inside the box, and the whiskers represent 10% and 90% measured values. Kaplan-Meier estimates and the risk of incident overall mortality ranked by quartile of (**B**) *p*-cresol sulfate and (**E**) indoxyl sulfate levels. Forest plots indicate the hazard ratio (95% CI) for incident (5 years) risks of overall mortality for (**C**) *p*-cresol sulfate and (**F**) indoxyl sulfate quartiles. Hazard ratio (unadjusted, black) and multivariable Cox model adjusted [gray; adjusted for age, sex, current smoking, high-density lipoprotein, low-density lipoprotein, triglyceride level, systolic blood pressure, diabetes mellitus, and high-sensitivity C-reactive protein (model 1) and model 1 + kidney function (model 2)]. Symbols represent hazard ratios and the 95% confidence intervals are indicated by line length.

**Fig 3:**
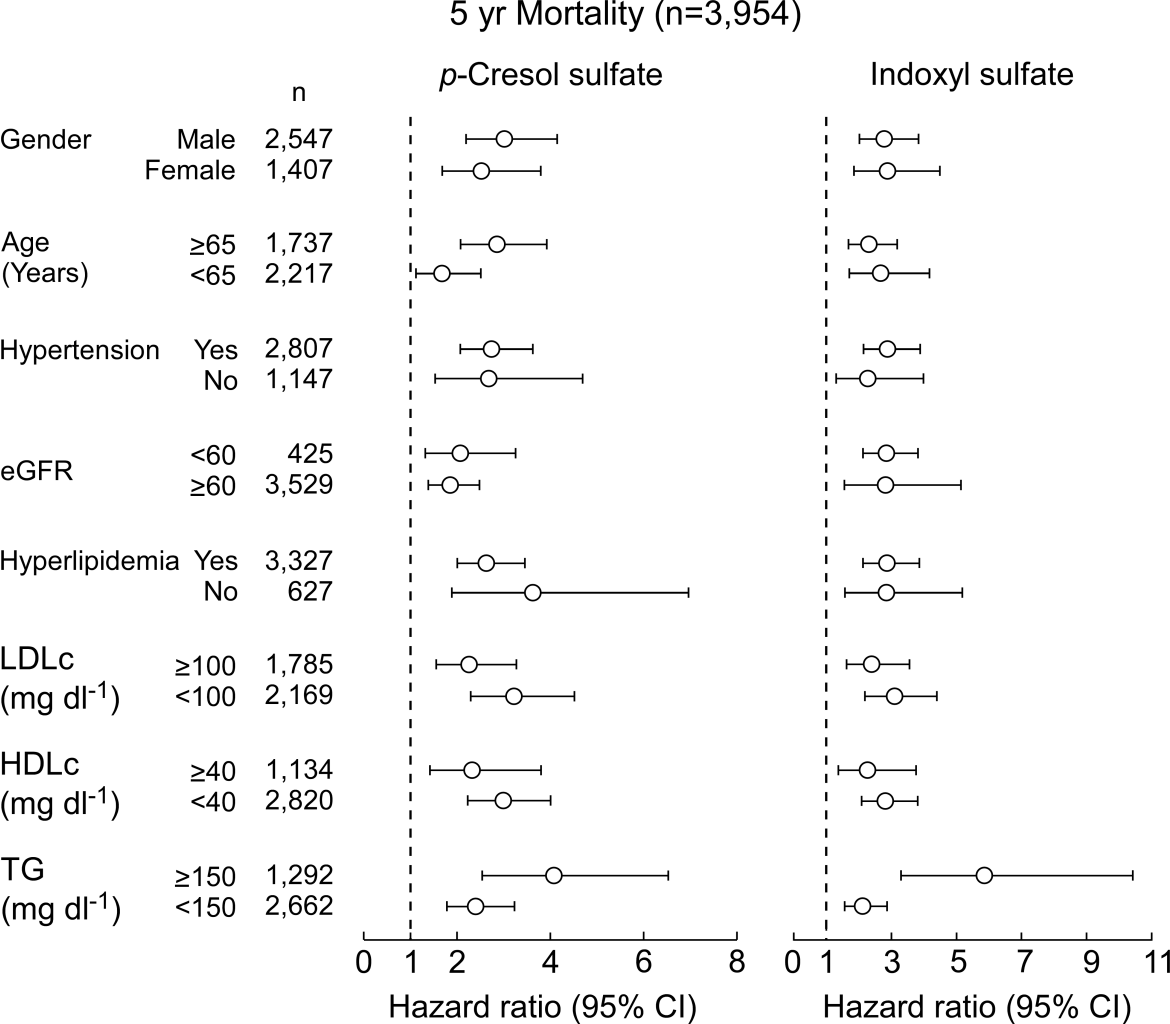
Long-term mortality risk among patient subgroups. Hazard ratio for 5-year overall mortality based on the Cox proportional hazards regression analysis comparing fourth vs first (referent) quartiles (**Q**). Data points (open circles) in the center indicate HR and 95% CIs are represented by line length. Numbers for each subgroup are indicated by n. eGFR, estimated glomerular filtration rate; LDL-c, low-density lipoprotein cholesterol; HDL-c, high-density lipoprotein cholesterol; TG, triglyceride.

We were somewhat surprised by the *p*-cresol finding because our GC-TOF methods employed were not optimized for detection of a volatile compound like *p*-cresol. Untargeted analyses as performed are not quantitative, and given use of a sample drying step where the analyte could be lost, we reasoned that the *p*-cresol detected using our analytical method most probably was a product of *p*CS hydrolysis during sample preparation (see Materials and Methods). Given the novelty of these findings (*p*CS previously has been linked to CVD and mortality risks, but only among those with significantly impaired renal function like end-stage renal disease and hemodialysis), we decided to validate our observations using quantitative methods in an independent (non-overlapping) cohort (*n* = 3,954 subjects; Table S2) using a complementary technique: targeted stable isotope-dilution LC-MS/MS (see Materials and Methods). We also expanded our targeted LC-MS/MS analyses to also include IS, since both *p*CS and IS are commonly referred to as “uremic toxins” and studied concurrently in clinical observational studies (17, 24–27). As shown in Fig. 2, both *p*CS and IS each were significantly higher in subjects who died over the course of 5 years (*P* < 0.001 each, Wilcoxon rank-sum test, Fig. 2A and D). Kaplan-Meier survival analyses revealed that subjects with either high *p*CS or IS levels had overall poorer survival over 5 years of follow-up (Fig. 2B and E). Specifically, individuals with *p*CS and IS levels in the highest vs lowest quartile (Q4 vs Q1) for each compound demonstrated a significantly increased risk of incident (5 years) death (HR = 2.83 [2.19–3.64]; *P* < 0.0001 and HR = 2.81 [2.16–3.65]; *P* < 0.0001, respectively). The associations between *p*CS and IS and death each remained significant after adjusting for traditional risk factors, as well as following additional adjustments for renal function (HR = 1.52 [1.16–2.00]; *P* = 0.002 and HR = 1.68 [1.27–2.21]; *P* = 0.0002 for *p*CS and IS, respectively) (Fig. 2C and F). Furthermore, the association between *p*CS or IS and risk of incident (5 years) death holds true when subjects were divided into subgroups of subjects with relatively preserved kidney function (estimated glomerular filtration rate [eGFR] ≥ 60 mL/min/1.73 m^2^) and subjects with impairment in kidney function (eGFR < 60 mL/min/1.73 m^2^) (Table S3). In further sensitivity analyses, we observed the associations between *p*CS and IS and mortality risk remained significant in both males and females alike, younger versus older, as well as within multiple different subgroups including subjects with or without hyperlipidemia or hypertension (Fig. 3). Collectively, these data demonstrate that *p*CS and IS, which are generally considered to be relevant only in the setting of renal disease, are associated with mortality in the broader population of individuals with predominantly preserved renal function, as well as in the absence of traditional cardiovascular risk factors.

### Identification of enzymes involved in *p*-cresol biosynthesis

**Fig 4:**
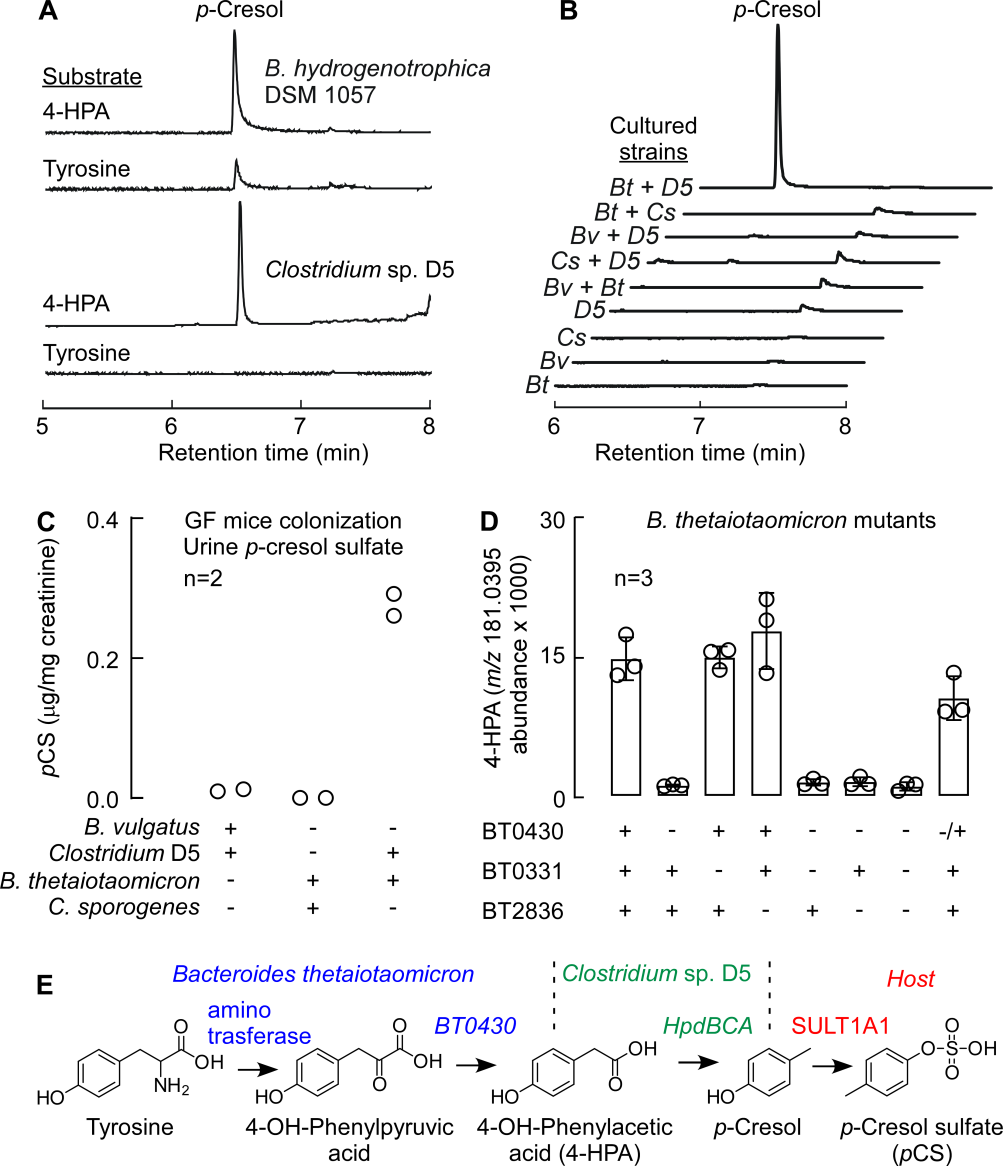
A collaborative pathway for the production of *p*-cresol. (**A**) GC-MS chromatogram of *p*-cresol production from metabolites of *Clostridium* sp. D5 and *B. hydrogenotrophica* DSM 10507 in the presence of 4-HPA and tyrosine (Tyr). (**B**) GC-MS chromatograms from mono-cultures and co-cultures. Bt, *B. thetaiotaomicron*; Bv, *Bacteroides vulgatus*; Cs, *Clostridium sporogenes*; D5, *Clostridium* sp. D5. (**C**) Urine *p*-cresol sulfate (*p*CS) from germ-free mice were co-colonized with either *B. vulgatus* and *Clostridium* D5 or *B. thetaiotaomicron* and *C. sporogenes* or *B. thetaiotaomicron* and *Clostridium sp*. D5. (**D**) The production of 4-HPA in *B. thetaiotaomicron* mutants. Data bars indicate the average LC-MS ion counts (negative mode) of three biological replicates ± SD. (**E**) Schematic of a collaborative pathway for the production of *p*-cresol and *p*-cresol sulfate.

Previous work from our laboratories and others had identified the bacterial genes required for indole production (21, 28), but the biosynthetic route for *p*-cresol is less understood. Several human gut commensals have initially been reported to produce *p*-cresol from tyrosine (29), but the quantity of *p*-cresol produced *in vitro* (typically 0.1–1.0 µg/mL) cannot account for the high levels of *p*CS commonly detected *in vivo* (20 mg/mL in CKD patients). Later, Saito et al. identified several *p*-cresol-producing commensals by screening multiple bacterial strains in culture media supplemented with tyrosine and its metabolites (30). Previous work showed that *hpdBCA* in *Clostridium difficile*, which encodes a multi-component 4-hydroxyphenylacetate (4-HPA) decarboxylase, can convert 4-HPA (31), and to a lesser extent tyrosine (32), to *p*-cresol. By searching for homologs of *hpdBCA* in reference genomes from the National Institutes of Health (NIH) Human Microbiome Project database, we found a similar operon in *Blautia hydrogenotrophica* DSM 10507 and *Clostridium* sp. D5. We tested the capacity of each strain to produce *p*-cresol from tyrosine and 4-HPA under anaerobic conditions. While both strains were able to produce *p*-cresol robustly from 4-HPA, production from tyrosine was negligible by *B. hydrogenotrophica* DSM 10507, consistent with previous findings (30), and undetectable by *Clostridium* sp. D5 (Fig. 4A). Given that metabolic byproducts excreted by one bacteria are often utilized by other bacteria in the gut microbial community (16, 33), an alternative hypothesis was suggested: the synthesis of *p*-cresol could be the result of a collaborative pathway involving two microbes, where the first microbe converts tyrosine to 4-HPA and a second decarboxylates 4-HPA to *p*-cresol.

We therefore performed studies to support the hypothesis that two microbes sequentially contributed to the overall tyrosine → *p*-cresol metabolic transformation within the gut. Certain common strains of *Bacteroides* have been reported to convert tyrosine to 4-HPA, including *B. thetaiotaomicron*, but not *Bacteroides vulgatus* (34). We therefore used these two strains in co-culture experiments in which we fed tyrosine to a pair of bacterial strains: the first strain was a 4-HPA-producing or non-producing *Bacteroides* species; and the second strain was *Clostridium* sp. D5, which converts 4-HPA to *p*-cresol, or *Clostridium sporogenes*, an *hpdBCA*-negative commensal. As shown in Fig. 4B, robust *p*-cresol production was only observed in the combination that included a 4-HPA producer and an *hpdBCA-*positive *Clostridium* (*B. thetaiotaomicron* and *Clostridium* sp. D5).

In pilot studies, we then colonized germ-free mice with the strain pairs tested *in vitro* for a week. As expected, robust production of *p*-cresol (leading to *p*CS in the urine) was only observed in mice colonized by *B. thetaiotaomicron* and *Clostridium* sp. D5. Mice colonized with *Clostridium* sp. D5 and *B. vulgatus* had low levels of *p*CS in their urine, indicating that *B. vulgatus* may produce small amounts of 4-HPA *in vivo*, or *Clostridium* sp. D5 may be able to produce *p*-cresol from tyrosine at a low level (Fig. 4C). Collectively, these data indicate that a collaborative pathway can account for *p*-cresol production *in vivo,* although we cannot exclude the possibility of an undiscovered strain that can convert tyrosine to *p*-cresol.

### Genes involved in 4-HPA production

Next, we sought to gain insight into the collaborative pathway by identifying the genes in *B. thetaiotaomicron* responsible for converting tyrosine to 4-HPA. By analogy to the conversion of phenylalanine to phenylacetate, we reasoned that the production of 4-HPA likely involved two steps: the transamination of tyrosine to 4-hydroxyphenylpyruvate (4-HPP) and the oxidative decarboxylation of 4-HPP to 4-HPA. A computational search of the *B. thetaiotaomicron* genome revealed >10 putative aminotransferases. Since aminotransferases often have overlapping substrate specificities (35), we reasoned that a single aminotransferase may not be responsible for the production of 4-HPA, so we focused instead on identifying the oxidative decarboxylase responsible for 4-HPA production.

In our previous study on *B. thetaiotaomicron*, we identified three homologs for 2-oxoacid ferredoxin oxidoreductase, a family of enzymes which decarboxylate pyruvic acid into the corresponding acetic acid derivative (36). The three homologs are coded by BT0332-329, BT0430-0429, and BT2836-2837 gene clusters. To determine which of these oxidoreductases contribute to 4-HPA production, we generated clean deletions of a single gene in each cluster and measured the capacity of each mutant to produce 4-HPA. Of the three single mutants, only Δ*BT0430* exhibited decreased 4-HPA production (Fig. 4D), and even this mutant produced residual levels of 4-HPA. Complementing the Δ*BT0430* mutant with BT0430 gene restored the production of 4-HPA to near-wild-type levels (Fig. 4D).

Strains harboring double and triple deletions of BT0430, BT0331, and BT2836 maintained low-level 4-HPA production without affecting bacterial growth, suggesting the existence of an additional pathway for 4-HPA production, potentially involving a member of the pyruvate:ferredoxin oxidoreductase (PFOR) superfamily (37). Nevertheless, our data suggest that BT0430 is predominantly responsible for 4-HPA production in *B. thetaiotaomicron*. Taken together, our findings support the collaborative pathway for *p*-cresol proposed in Fig. 4E.

### Engineering *B. thetaiotaomicron* to produce indole and *p*-cresol simultaneously

**Fig 5:**
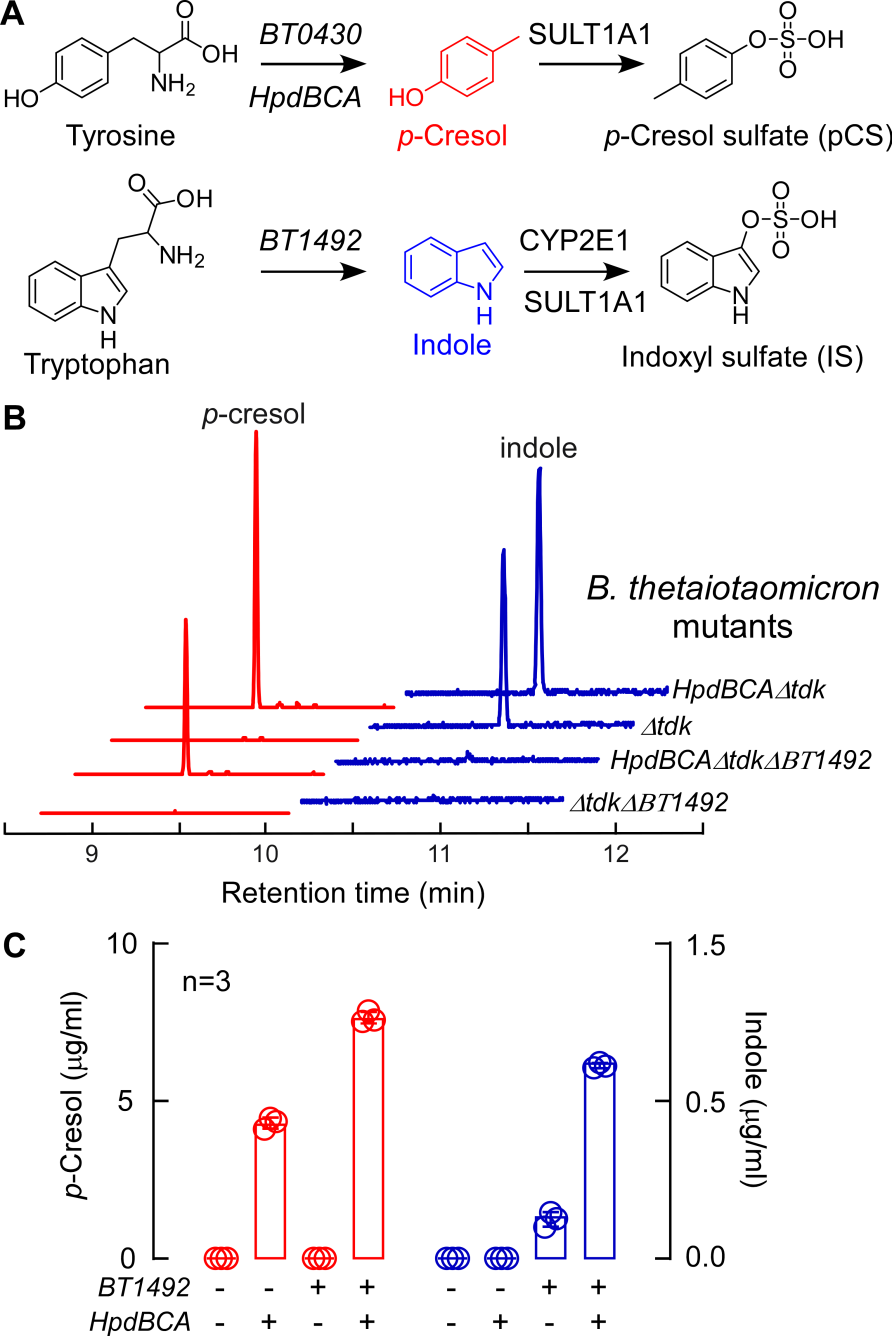
Engineered *Bacteroides* strains synthesize *p*-cresol and indole *in vitr*o. (**A**) Schematic presentation of genes involved in *p*-cresol and indole production. (**B**) Representative GC-MS chromatograms of selected ions for *p*-cresol (red; *m*/*z* 107.1 selected ion) and indole (blue; *m*/*z* 117.1 selected ion) production in culture from engineered *B. thetaiotaomicron* strains. Strains with the *hpdBCA* operon produce *p*-cresol, whereas strains containing the *BT1492* gene produce indole. (**C**) Amounts of *p*-cresol (red) and indole (blue) produced by cultured *B. thetaiotaomicron* mutants (*n* = 3) measured by GC-MS.

Indole and *p*-cresol are both connected to the potential cardiovascular sequelae of CKD and end-stage renal disease, and each metabolite is correlated with mortality risk in humans (even with preserved renal function). Since these metabolites are produced simultaneously by the microbiome, we sought to create a system that would enable us to study their effects on the host. Such a system could be complex and difficult to control if it involves multiple bacterial species. As an alternative approach, we set out to engineer a single strain of *B. thetaiotaomicron* to produce either *p*-cresol or indole alone, or both metabolites simultaneously (Fig. 5A).

We chose *Bacteroides thetaiotaomicron* VPI-5482 as our chassis for engineering; this strain harbors an indole-producing tryptophanase (*BT1492*+) but no operon for *p*-cresol biogenesis (*hpdBCA*). We started with *B. thetaiotaomicron* ∆*BT1492*, a tryptophanase mutant previously generated in our lab (21); we refer to this indole^−^/*p*-cresol^−^ strain as Δ*tdk*Δ*BT1492*. We transferred the *hpdBCA* operon from *C. difficile* JIR 8094 into this strain under the control of a constitutive promoter derived from *Bacteroides* phage B124-14 (38), yielding the indole^−^/*p*-cresol^+^ strain *HpdBCA*Δ*tdk*Δ*BT1492*. Since the level of indole production from wild-type *B. thetaiotaomicron* is low, we inserted two additional copies of BT1492 (three total) to generate the indole^+^/*p*-cresol^−^ strain. Finally, we constructed the indole^+^/*p*-cresol^+^ strain *HpdBCA*Δ*tdk*, which harbors two additions: the *hpdBCA* operon from *C. difficile* and one additional copy of BT1492 (two total). These strains were tested for their capacity to produce *p*-cresol and indole *in vitro*. As predicted, the presence of *hpdBCA* and *BT1492* determined the production of *p*-cresol and indole, respectively (Fig. 5B and C). Notably, the fact that an *hpdBCA* operon from *Clostridium* functions in *B. thetaiotaomicron* suggests that *Bacteroides*—a Gram-negative that is the most common genus of gut bacteria—could be a model host for studying a broader range of metabolites produced exclusively by the Gram-positive *Firmicutes*.

### *hpdBCA* and *BT1492* modulate thrombosis potential *in vivo*

Previous studies have shown that pharmacologic provision (i.e., added exogenously) of *p*CS or IS can promote pro-thrombotic phenotypes (28, 39, 40), but not in the context of direct demonstration of heightened host thrombosis by a defined gut microbial enzyme system. We therefore set out to address this gap in knowledge using the strains of *Bacteroides* engineered to produce individually either *p*CS, IS, or both metabolites. First, we validated an *in vivo* assay for thrombosis induced by arterial injury that was modulated by direct provision of the uremic toxins (Fig. S1A). After raising the levels of *p*CS and IS by injecting them as purified chemicals intraperitoneally, an FeCl_3_-induced carotid artery injury model was used to model thrombosis. We measured two parameters: the rate of platelet clotting following carotid artery injury, and the time to cessation of flow within the carotid artery. *p*CS and IS each individually induced heightened platelet thrombus formation within the injured carotid artery (Fig. S1A) and reduced the time to cessation of blood flow following injury (i.e., the occlusion time) when compared to mice treated with saline, the vehicle (Fig. S1B; *P* < 0.0001, Kruskal-Wallis).

**Fig 6:**
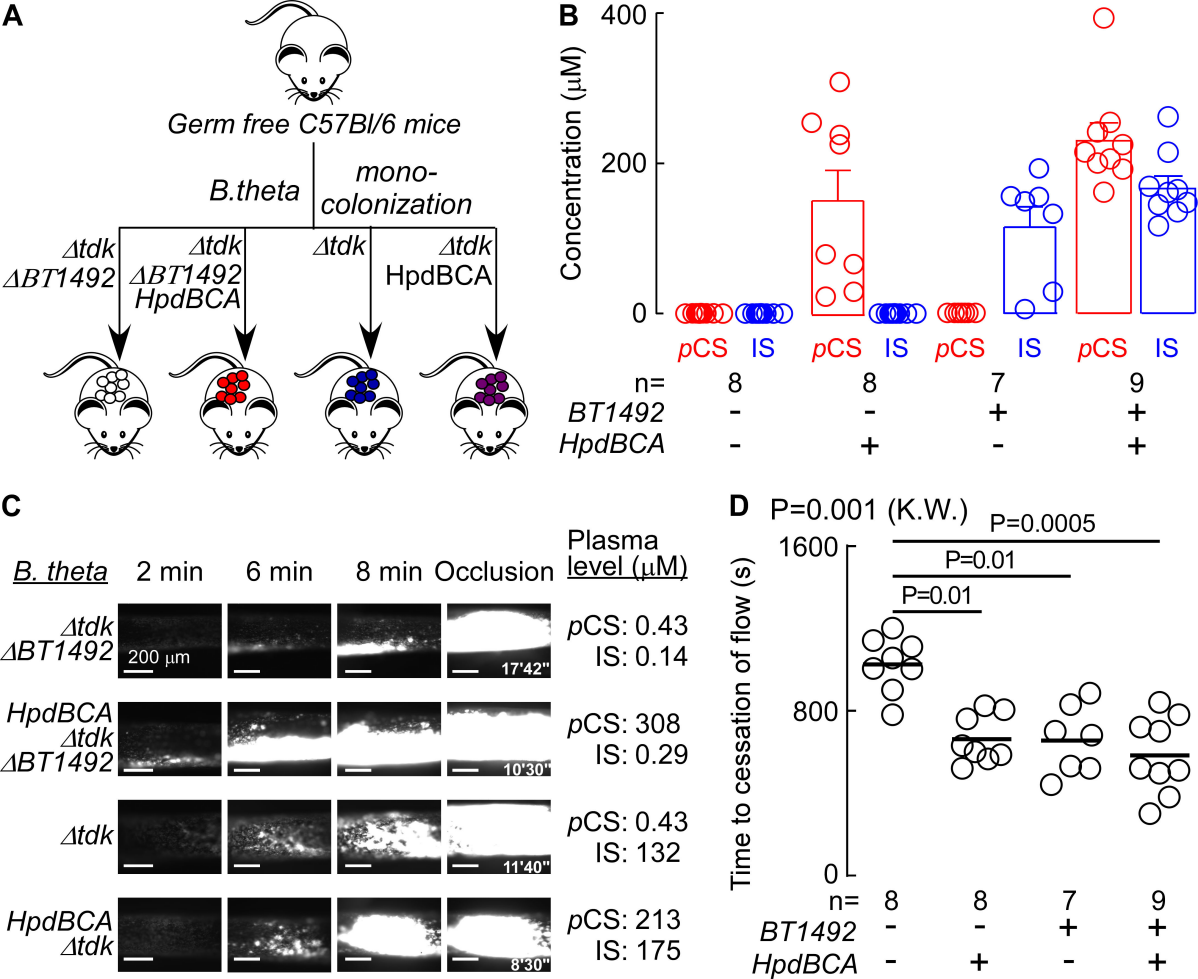
Difference in microbial genes responsible for *p*-cresol or indole production is associated with increased *in vivo* thrombosis potential. (**A**) Scheme illustrating microbial transplant study design. Germ-free (C57B1/6) mice were subjected to gavage with four different engineered *B. thetaiotaomicron* strains with different capacities for *p*-cresol and indole production: (Δ*BT1942* (white), *hpdBCA ΔBT1942* (red), Δ*tdk* (blue), and *hpdBCA* Δ*tdk* (purple). (**B**) Levels of *p*CS and IS in mouse plasma 2 days post gavage and 24 h post folic acid intraperitoneal (IP) injection at time of thrombosis model. (**C, D**) *In vivo* thrombosis potential was measured by the FeCl_3_-induced carotid artery injury model. Representative vital microscopy images of carotid artery thrombus formation are shown at the indicated time points following arterial injury (**C**), and time to cessation of blood flow in mice (**D**) measured in the indicated number of animals (*n* = 7–9). The bar represents mean time to cessation of blood. Significance was measured with a Kruskal-Wallis (K.W.) test followed with Dunn’s multiple comparisons test.

In a subsequent series of experiments, we tested whether the engineered strains of *Bacteroides* both contribute to *p*CS and IS production and foster a pro-thrombotic phenotype when transplanted into a mammalian host. We thus colonized germ-free mice with the four strains of *B. thetaiotaomicron* (*BT1492*/*hpdBCA* +/+, +/−, −/+, −/−) for up to 7 days and then measured both colonization efficiency and plasma levels of *p*CS and IS. Notably, *the B. thetaiotaomicron* mutants with different capacities for *p*-cresol and indole production did not significantly impact colonization level (Fig. S2). However, the status of *BT1492* and *hpdBCA* determined the level of IS and *p*CS, respectively, demonstrating that our genetically engineered strains enable us to control the presence/absence of each metabolite independently, recapitulating the entire metaorganismal pathways (Fig. 6A and B). Finally, we tested whether the presence of a functional *hpdBCA* or *BT1492* gene product within colonized gnotobiotic mice can modulate platelet function and thrombosis potential *in vivo*. Germ-free mice colonized by each strain were subjected to arterial injury. We observed that the rate of thrombus generation increased, and the time to cessation of blood flow within the injured vessel decreased, in mice harboring commensals with either a functional *hpdBCA* or *BT1492* in their gut colonist (Fig. 6C and D). These results demonstrate that bacterially produced indole or *p*-cresol, converted to IS and *p*CS, respectively, by the host, each are capable of increasing thrombosis potential in a well-controlled setting. Notably, the presence of both (combined) loci (*BT1492+*/*hpdBCA+*) did not exacerbate the pro-thrombotic phenotype.

### Elevated levels of *tryptophanase* and *hpdBCA* gene homologs are independently associated with atherosclerotic cardiovascular disease (ASCVD)

**Fig 7:**
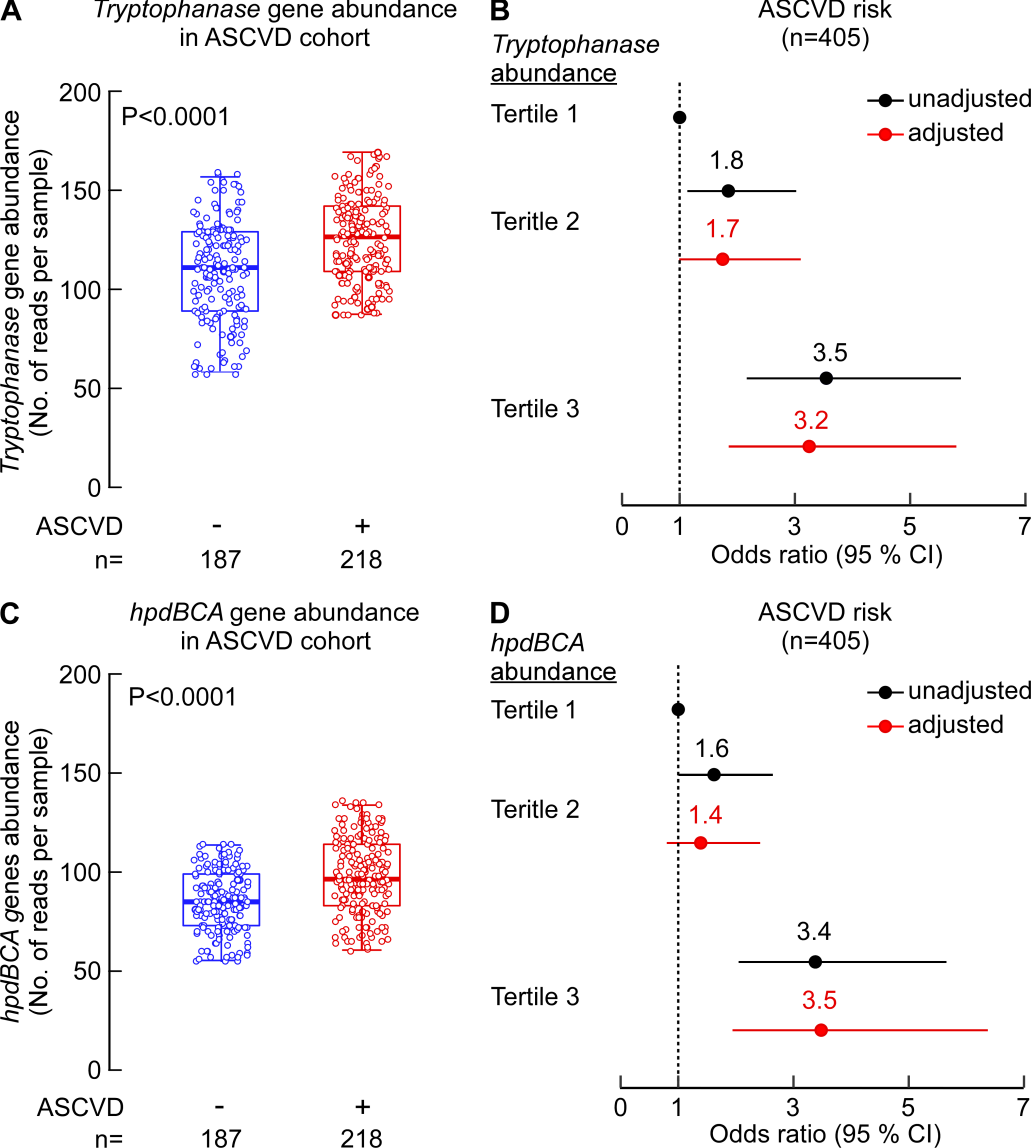
Elevated levels of *tryptophanase* and *hpdBCA* gene homologs are associated with ASCVD. The metagenomics data from Jie at al.’s study (41) were used to determine fecal abundance of tryptophanase and *hpdBCA* genes in individuals with (*n* = 218) and without ASCVD (*n* = 187). (**A, C**) Box-whisker (5%–95%) plots of *tryptophanase* (**A**) and *hpdBCA* (**C**) gene abundance in the fecal metagenome of control individuals vs individuals with ASCVD. *P*-values were calculated using Wilcoxon rank-sum test. (**B, D**) Forest plots indicating the ASCVD prevalence according to the tertiles of *tryptophanase* (**B**) and *hpdBCA* (**D**) gene abundance. The multivariable logistic regression model for odds ratio in panels **B** and **D** included adjustments for age, sex, and hyperlipidemia. The 95% CI is indicated by line length.

Next, we looked at the abundance of *tryptophanase* and *hpdBCA* genes in a previously published metagenomics analysis on stool samples from a cohort composed of individuals with ASCVD (*n* = 218) versus controls (*n* = 187) (41). As shown in Fig. 7, individuals with ASCVD had higher abundances of *tryptophanase* and *hpdBCA* genes when compared to controls (*P* < 0.0001 for both genes, Fig. 7A and C). The same trend (higher in ASCVD vs controls) was observed when *tryptophanase* and *hpdBCA* gene expressions were each normalized to median expression level of six different single copy microbial ribosomal reference genes, as outlined in Materials and Methods (*P* = 0.012 and *P* = 0.016, respectively, for *tryptophanase* and *hpdBCA*). Subsequent analysis showed subjects with elevated levels of either *tryptophanase* or *hpdBCA* genes (third tertile [(T3]) had higher prevalence of ASCVD compared to those with low (first tertile [T1]) levels, even after adjustment for traditional CVD risk factors (odd ratio [OR] 95% CI for ASCVD, OR = 3.2 [1.9–5.8], *P* < 0.0001 and OR = 3.5 [1.9–6.4], *P* < 0.0001, respectively) (Fig. 7B and C).

## DISCUSSION

Patients with chronic kidney disease or end-stage renal disease are known to have elevated levels of *p*CS and IS, both of which are also poorly dialyzable. Moreover, the association of these “uremic toxins” with CVD and mortality risk in patients with impaired renal function has been reported in multiple studies (17–19, 42). As a consequence, *p*CS and IS have been studied almost exclusively in the context of kidney disease. By using untargeted metabolomics as a discovery platform on individuals with primarily preserved renal function, we unexpectedly observed that *p*-cresol (presumed proxy for *p*CS) and mortality risk might be extended to the much broader population of individuals with preserved kidney function. This led to studies using a separate validation cohort (*n* = 3,954) and quantitative stable isotope labeled LC-MS/MS analyses that confirmed that the uremic solutes (both *p*CS and IS) are associated with heightened mortality risks outside the setting of renal disease. It is also notable that in a recent multi-center, randomized, controlled, isocaloric protein intervention trial conducted in subjects (*n* = 151) with diabetes and preserved kidney function, a 12-week high-protein diet was shown to increase circulating levels of IS, but not pCS (43).

*p*CS is among the most prevalent and concentrated of circulating microbiome-derived metabolites (with average high circulating values of 15 µM in normal and 230 µM in uremic subjects [20]), yet its biosynthetic pathway was only partially characterized. By showing that *p*-cresol can be produced collaboratively by certain *Bacteroides* species (tyrosine → 4-HPA transformation) in co-culture with *hpdBCA-*positive *Firmicutes* (4-HPA → *p*-cresol transformation), we complete a plausible, genetically defined pathway to *p*-cresol. Our recent study showed that another gut microbial prevalent enzyme, phenylpyruvate decarboxylase (PPDC), can also catalyze the 4-hydroxypyruvic acid conversion into 4-HPA (37). Consequently, future studies will be needed to determine the actual contribution of each of these two enzymes (phenylpyruvate:ferredoxin oxidoreductase [PPFOR] and PPDC) in the formation of intestinal 4-HPA. In subsequent experiments, we engineered the second half of the *p*-cresol pathway into *B. thetaiotaomicron,* creating a simple system in which the production of two metabolites can be easily controlled and studied. We propose that a similar approach in the future could be used to de-orphan the pathways for hundreds of microbiome-derived metabolites and study their effects on the host.

It has previously been described that IS increases thrombotic risk through enhancement of platelet activity (44), by altering tissue factor stability (45) and by enhancing thrombus formation in animal models of vascular injury (28, 44). Elevated levels of *p*CS, on the other hand, promoted vascular dysfunction and remodeling (39), while both solutes exerted proinflammatory effects that have been proposed to promote vascular damage (40). Here, we showed that gut microbial activity of *hpdBCA* and *tryptophanase* genes, individually or in tandem, was sufficient to impact host thrombosis potential. Moreover, the present studies show for the first time that fecal abundances of both microbial genes, *hpdBCA* and *tryptophanase,* were independently associated with ASCVD in humans.

The present studies, and previous work in which other pro-thrombotic gut microbiota-generated metabolites are identified (e.g., trimethylamine N-oxide [TMAO], phenylacetylglutamine [PAGln] [11, 12, 46]) and pathways responsible for their production (36, 37, 47, 48), provide a platform for developing new therapeutic approaches to potentially attenuate thrombosis (and potentially broader CVD-related phenotypes) in the host. Importantly, while the physiologic effect of these metabolites can be the same (enhanced thrombosis and platelet hyper-responsiveness), this approach will allow personalized treatments tailored to the particular set of pathways present and active in each individual. We anticipate a future where individually tailored therapeutic interventions to reduce CVD risks and thrombosis potential are driven by the pattern of elevated pro-thrombotic gut microbial metabolites a subject possesses. As a proof of concept of the idea of small-molecule inhibition of a gut microbial pathway to elicit beneficial effect within the host, we recently demonstrated that a non-lethal inhibitor of the gut microbial enzyme catalyzing conversion of choline to trimethylamine (TMA)/TMAO (CutC) inhibited both atherosclerosis (48) and thrombosis potential *in vivo (49)*.

Numerous studies have reported that changes in the gut microbial community composition are associated with a variety of diseases including CVD, renal disease, and numerous others (5, 10, 50, 51). Identifying microbial pathways responsible for the production of individual bioactive metabolites that impact the host, both harmful and beneficial, could provide a basis for designing communities that produce a suite of compounds beneficial to the host. While linking microbial products to human diseases, elucidating their biological effects, and identifying microbes and enzymes involved in their metabolism, this study and other recent work (12, 16, 21, 36, 37, 46, 49, 52–57) demonstrate that integrating clinical and mechanistic studies with bioengineering technologies, and targeted inhibition of gut microbial enzymes, is a logical path forward toward reaching a future where personalized therapeutic interventions to reduce cardiovascular or metabolic disease risks are achievable.

## MATERIALS AND METHODS

### Human subjects

Both the discovery cohort (*n* = 1,149) employed for untargeted metabolomics investigations and the independent non-overlapping validation cohort (*n* = 3,954) were obtained from sequential consenting subjects enrolled in the study GeneBank at the Cleveland Clinic: Molecular Determinants of Coronary Artery Disease (GATC). GeneBank is registered under ClinicalTrials.gov Identifier: NCT00590200. Fasting plasma samples were collected in EDTA tubes and immediately processed and stored at −80° C until analyzed. Adjudicated all‐cause mortality was monitored for 5 years. High-sensitivity C-reactive protein, serum creatinine, lipid profiles, and hemoglobin A1C levels were measured on the Roche Cobas platform (Roche Diagnostics).

### Mice

Animal care and experimentation were consistent with NIH guidelines. C57BL/6J mice, 8–10 weeks old, were purchased from The Jackson Laboratory and maintained in facilities at the Cleveland Clinic Lerner Research Institute. Some of the studies employed germ-free C57BL/6 male mice bred at the University of Wisconsin-Madison gnotobiotic animal facilities and shipped germ-free to the Cleveland Clinic Lerner Research Institute Gnotobiotic Facility. Studies in Fig. 3 were performed using germ-free C57BL/6 mice from the Mazmanian Laboratory GF colony at California Institute of Technology.

### Bacterial strains and culture conditions

All bacterial strains and plasmids used in this study are shown in Tables S4 and S5. All *Bacteroides* were cultured in brain heart infusion (BHI) agar medium supplemented with 10% horse blood, *Bacteroides* TYG (tryptone-yeast extract-glucose) broth, or minimal medium (MM). All *Clostridium* strains were cultured in *Clostridium* TYG (3% wt/vol tryptone, 2% wt/vol yeast extract, 0.1% wt/vol sodium thioglycolate) broth or MM at 37°C in anaerobic chamber. *Blautia hydrogenotrophica* DSM 10507 was cultured in peptone yeast glucose (PYG) broth (Recipe #1139 on Recommended Media for Microorganisms list in DSMZ). *Escherichia coli* strains were aerobically cultured in Luria-Bertani (LB) broth. When appropriate, 100 µg/mL carbenicillin, 25 µg/mL erythromycin, 200 µg/mL gentamicin, and 200 µg/mL 5-fluoro-2’-deoxyuridine were supplemented into the medium.

### Untargeted GC-MS analysis of human plasma samples

Untargeted gas chromatography mass spectrometry analyses of human plasma samples were performed as previously reported (58). ChromaTOF (Leco) software was used for data acquisition. Raw data files were processed using the metabolomics BinBase database (59). All database entries in BinBase were matched against UC Davis metabolomics center’s mass spectral library. *p*-Cresol was detected as trimethylsilyl (TMS) derivative (fatty acid methyl ester [FAME] retention index 280360) and *m*/*z* 165 was used as quantification ion.

### Targeted LC-MS/MS analysis of human plasma samples

Targeted stable isotope-dilution liquid chromatography with on-line electrospray ionization tandem mass spectrometry (LC-MS/MS) was used for quantification of *p*CS and IS as previously described (60).

### Gene disruption in *B. thetaiotaomicron*

All PCR amplifications were done with PrimeSTAR Max DNA polymerase (Takara Bio) according to the manufacturer’s instructions. Sequences of primers are shown in Table S6. To construct target gene deletion mutants, a previously described double-crossover recombination method was used (61).

For complementation of the *BT0430* deletion, the *BT0430* gene was amplified and assembled with pNBU2-*bla-ermG*b under control of promoter from the sigma 70 (BT1311) of *B. thetaiotaomicron* VPI 5482 (62) using Gibson Assembly Kit (New England Bio) to yield pMFT01. *Escherichia coli* S17-1 λ *pir* competent cells were transformed with the assembled plasmids by electroporation and transformants were confirmed by PCR. The positive clone harboring assembled plasmid was cultivated, prepared, and sequenced. The correct plasmid was introduced into *B. thetaiotaomicron* ∆*tdk* ∆*BT0430* by conjugation, and erythromycin-resistant strains were selected followed by PCR confirmation.

### Overexpression of *BT1492* and *hpdBCA* in *B. thetaiotaomicron*

Vector maps used in this study for overexpression are shown in Fig. S2. *BT1492* was amplified by PCR and assembled with pNBU2_*bla_ermGb* and pNBU2_*bla_tetQb* under the phage promoter from *Bacteroides* phage B124-14 using Gibson Assembly kit to make pMFT03 and pMFT04. The *hpdBCA* operon (gene ID: 640155897, 640155898, 640155899) from *C. difficile* JIR8094 was also amplified by PCR and assembled with pNBU2_*bla_ermGb* under the phage promoter to yield pMFT02. *Escherichia coli* S17-1 λ *pir* competent cells were transformed with these assembled plasmids by electroporation, and transformants were confirmed by PCR. The positive clone harboring assembled plasmid was cultivated, and plasmid was prepared and verified by sequencing. The plasmid was introduced and integrated into *B. thetaiotaomicron* ∆*tdk* or *B. thetaiotaomicron* ∆*tdk ∆BT1492* by conjugation, and appropriate antibiotic-resistant strains were selected followed by PCR confirmation. For the strain expressing *hpdBCA*, pMFT02 was integrated into *B. thetaiotaomicron* ∆*tdk* ∆*BT1492* by conjugation. For the strain expressing *BT1492*, pMFT03 and pMFT04 were integrated into *B. thetaiotaomicron* ∆*tdk* sequentially. For the strain expressing *HpdBCA* and *BT1492*, pMFTC02 and pMFTC04 were integrated into *B. thetaiotaomicron* ∆*tdk* sequentially.

### Extraction of metabolites from bacterial culture medium

For metabolite analysis of *Clostridium* sp. D5 and *B. hydrogenotrophica* DSM 10507, they were cultured anaerobically in rich medium overnight, as described above. Cells were harvested and washed twice with MM. The pellets were re-suspended in MM with 0.1 mg/mL tyrosine or 0.1 mg/mL 4-HPA at a final optical density at 600 nm (OD_600_) of 1.0 and incubated for 24 h. Cell suspensions were extracted with equal amount of ethyl acetate and organic layers were analyzed by GC-MS.

For metabolite analysis of *Bacteroides* strains in mono-culture, strains were grown in rich medium overnight and the cells were harvested followed by MM wash. The cell pellet was re-suspended with MM containing 0.5 mg/mL tyrosine (for *p*-cresol production) or tryptophan (for indole production) to a final OD_600_ of 1.0 and incubated anaerobically for 24 h. For *p*-cresol and indole detection, the cultures were extracted with ethyl acetate and analyzed by GC-MS as outlined below. For 4-HPA detection, cells were extracted with acetone (20% in final) and supernatants after centrifugation were analyzed by LC/MS.

For metabolite analysis of co-culture experiment, *Bacteroides* strains and *Clostridium* strains were cultivated anaerobically in rich medium overnight and cells were harvested and washed with MM. The cell pellets were re-suspended in MM containing 0.5 mg/mL tyrosine to OD_600_ of 1.0 and combined. After 24 h incubation, samples for GC-MS analysis were prepared as described above.

### Analysis of bacterial culture metabolites and mouse urine

*p*-Cresol and indole were analyzed with a split ratio of 10:1 using a GC-MS (Agilent 7890 GC coupled to an Agilent 5,977 MSD) with an HP-5MS fused silica capillary column (30 m × 250 µm × 0.25 µm, Agilent) or DB-WAXetr capillary column (30 m × 250 µm × 0.25 µm, Agilent) with helium as carrier gas, injector temperature of 250°C with non-linear temperature gradient between 40°C and 320°C; *m/z* 107.1 and 117.1 were used as quantifying ions for *p*-cresol and indole, respectively. 4-HPA, *p*CS, and IS were analyzed by an LC-MS/MS system (Agilent 1260 coupled to an Agilent 6120 quadrupole) or an liquid chromatography time-of-flight (LC-TOF) system (Agilent 1290 LC system coupled to an Agilent 6530 QTOF) with a UK-C18 column (3 µm, 4.6 × 75 mm Unison, Imtakt). A discontinuous gradient employing a mixture of 10 mM ammonium acetate with 0.1% formic acid (solvent A) and acetonitrile with 0.1% formic acid (solvent B) was used for chromatographic separation; *m*/*z* 151.0401, 187.0071, and 212.0023 in negative ion mode were used for measuring 4-HPA, *p*CS, and IS, respectively. Concentration of creatinine was measured using Colorimetric Creatinine Assay Kit (Abcam) according to manufacturer’s instructions.

### Gnotobiotic mouse colonization

#### Co-colonization of *Bacteroides* and *Clostridium* sp*.* D5

Stationary phase liquid cultures of *Clostridium* and *Bacteroides* spp. were combined at a 1:1 (vol/vol) ratio while maintained in an anaerobic chamber and sealed in airtight tubes until administration to mice. Germ-free C57Bl/6 mice (6 weeks old) from the Mazmanian Laboratory GF colony were gavaged with 100 µL of the combined culture and strictly maintained in autoclaved microisolator cages. One week following colonization, urine samples were collected passively during brief restraint of mice by hand and analyzed for metabolite levels as described above. A normalized mass of fecal material from each mouse was re-suspended in BHI medium and plated on brain heart infusion supplemented with yeast extract (BHIS) plates. Visual observation confirmed equivalent levels of colony morphologies of both the respective *Bacteroides* and *Clostridium* spp. across all plates and bacterial pairs. PCR of fecal pellets from these mice also showed accurate colonization. This is now included in the revised manuscript.

#### Mono-colonization with wild-type and engineered strains of *B. thetaiotaomicron*

*B. thetaiotaomicron* mutants were grown on tryptic soy blood agar plates (Anaerobe Systems) anaerobically for 48–72 h at 37°C. Single colonies were picked and used to inoculate Mega Medium (3 mL) in prepared hungate tubes. Cultures were grown anaerobically for 18–24 h at 37°C. At that time, an aliquot of culture (500 µL) was removed and the remaining bacterial culture was diluted 1:1 with glycerol (40%) in water (vol:vol) and stored at −80°C. Germ-free, C57BL/6, male, 8- to 10-week-old mice were mono-colonized by oral gavage with ~0.2 mL of bacterial culture inside the biological safety cabinet, using the indicated mutants. Mice were maintained on a sterilized diet and, 24 h prior to *in vivo* thrombosis, were injected with filter sterilized folic acid (250 mg/kg) to induce kidney injury and increase metabolites levels. At the time of sacrifice (~7 days post colonization), mice were subjected to carotid artery FeCl_3_ injury thrombosis assay and tissues were collected immediately after the assay, frozen, and stored at −80°C. Following colonization, the investigators were not blinded from treatment groups to avoid cross contamination.

To confirm colonization, DNA was isolated from flash frozen cecal contents of colonized mice with the NucleoSpin Tissue kit (Macherey-Nagel) according to manufacturer’s instructions for bacterial DNA isolation. Isolated DNA was used in a PCR reaction with GoTaq Green Master Mix and the 8F and 1492R 16S rRNA universal primers. PCR reactions were carried out in a 96-well plate with 20 µL final reaction volumes as follows: 95°C for 2 minutes; 95°C for 30 seconds, 51°C for 30 seconds, 72°C for 1 minute and 20 seconds (×30); 72°C for 5 minutes. Completed reactions were sent to Eurofins Genomics for PCR cleanup and 16S rRNA gene sequencing using their standard house primers (16F). Sequence identity was confirmed using NCBI BLAST (Basic Local Alignment Search Tool developed by the National Center for Biotechnology Information). To determine cecal bacterial load counting, a portion of the frozen ceca were weighed, thawed under anaerobic conditions, and suspended in PBS buffer. Dilutions of the cecal suspensions were then plated on BHI agar plates, incubated for 72 h at 37°C under anaerobic conditions for colony counting.

### Thrombosis assay

Monocolonized or i.p. injected mice (vehicle [normal saline]; *p*CS [50 mg/kg]; IS [50 mg/kg]) were anesthetized with ketamine and xylazine and subjected to a carotid artery injury, and both the rate of thrombus generation and blood flow monitored in real time by fluorescence intravital microscopy with continuous image capturing as previously described (11). Briefly, rhodamine 6G (100 µL; 0.5 mg/mL) was injected directly into the right jugular vein to label platelets. The left carotid artery was exposed and injured by placing 1.5 × 1.5 mm^2^ Whatman filter paper soaked in 10% FeCl_3_ solution to the surface of the vessel for 1 minute. After removing the paper, the vessel was covered with saline. Thrombus formation was observed in real time using intravital fluorescence microscopy equipped with video recording. Time to cessation of blood flow through clot formation for all studies was determined by visual inspection by two independent investigators.

### Metagenomic analyses of *tryptophanase* and *hpdBCA* gene abundances and prevalent ASCVD

We used the publicly available metagenomics data from a previously published ASCVD case/control cohort (41). ASCVD subjects (*n* = 218) were Han Chinese, 40–80 years old with stable angina, unstable angina, or acute myocardial infarction (AMI). ASCVD diagnosis was confirmed by coronary angiography and defined as ≥50% stenosis in single or multiple vessels were included. Han Chinese Controls (*n* = 187) comprised of subjects free of clinically evident ASCVD symptoms at the time of the medical examination.

#### Custom reference database creation

The Entrez Programming Utilities (E-utilities: https://www.ncbi.nlm.nih.gov/books/NBK25501/) implemented in the eutils package (https://github.com/biocommons/eutils) were used to retrieve all microbial protein-coding sequences (keywords: -db protein -query "(gene name) AND "bacteria"[porgn: txid2]" | efetch -format fasta_cds_na nucleotides) from NCBI. The protein-coding sequences (nucleotides) provided by NCBI’s protein database were compared to protein-coding sequences in the MGnify database (63) using blastn (64). Using a minimum identity and length cutoff of 90% and 80%, a tabular output was generated from blastn output using R (https://github.com/bioinfo-core-BGU/parse_blast). The MGnify database’s homologs were combined with NCBI sequences to generate a custom microbial protein-coding sequence database.

#### *De novo* metagenome assembly and screening

Shotgun microbiome data from the ASCVD cohort (41) were quality filtered and trimmed using the nesoni pipeline (https://github.com/Victorian-Bioinformatics-Consortium/nesoni). Quality-filtered reads were assembled into contigs using a megahit assembler (65). Metagenome assemblies were screened for possible mis-assembly events using metaMIC pipeline (66). Quality-filtered metagenome contigs were annotated using the prokka pipeline (67). Protein-coding genes from individual metagenome assemblies were compared against our customized reference database using blastn (minimum identity = 90% and minimum length = 80%) 2. To quantify the abundance of protein-coding genes, quality-filtered metagenome reads were mapped on the protein-coding genes that showed hits to our custom reference database using vsearch (68). Abundance (counts) patterns of *hpdBCA* and *tryptophanase* gene were used for differential abundance and metadata association analyses. Relative abundance of *rplB*, *rplC*, *rpsS*, *rplD*, *rplE*, and *rpsQ* genes was used to normalize the gene count matrix.

### Statistical analyses

Student’s *t*-test (two tailed) or Wilcoxon’s rank-sum test for continuous variables and χ^2^ test for categorical variables were used to examine the differences between groups. Rank-based nonparametric Kruskal-Wallis test was used for non-normally distributed data. In the box-whisker plot, the upper and lower boundaries of the box represent the 25th and 75th percentiles, the median is marked by a horizontal line inside the box, and whiskers represent 10% and 90% of relative measured values. Categorical data are presented as *n* (%). HR for death at 5-year follow-up and corresponding 95% CI were estimated using both univariable (unadjusted) and multivariable (adjusted) Cox models. Kaplan-Meier analysis with Cox proportional hazards regression was used for time-to-event analysis to determine HR and 95% CI for 5 year mortality. Adjustments were made for individual traditional cardiac risk factors including age, sex, high-density lipoprotein, low-density lipoprotein, triglycerides, current smoking, diabetes mellitus, systolic blood pressure, and high-sensitivity C-reactive protein level (model 1) and all factors form model 1 and CKD (define as eGFR <60 mL/min/1.73 m^2^) (model 2). To further test for the relationship between *tryptophanase* and *hpdBCA* gene abundance and ASCVD, odds ratio for binary ASCVD and corresponding 95% CI were calculated using both univariable (unadjusted) and multivariable (adjusted) logistic regression models with cohort adjustments made for age, sex, and dyslipidemia. All data are presented as mean ± standard deviation or SEM or median with interquartile range. Statistical tests used to compare conditions are indicated in figure legends. GraphPad PRISM versions 8.0 and R 3.4.2 (Vienna, Austria, 2017) were used for generation of graphs and statistics.

## Data Availability

All source data for figures included in the manuscript were deposited in Zenodo repository (https://zenodo.org/record/8338175). There are restrictions to the availability of some of the clinical data generated in the present study because we do not have permission in our informed consent from research subjects to share data outside our institution without their authorizations. Under these situations, data shared is in summary format.

## SUPPLEMENTAL MATERIAL

Supplemental Material mbio.01331-23-s0001.pdf Tables S1-S6; Fig. S1-S3.

ASM does not own the copyrights to Supplemental Material that may be linked to, or accessed through, an article. The authors have granted ASM a non-exclusive, world-wide license to publish the Supplemental Material files. Please contact the corresponding author directly for reuse.
