# Supplementary material for: Microbe-derived uremic solutes enhance thrombosis potential in the host
Source: mBio. 2023 Nov 10;14(6):e01331-23. doi: 10.1128/mbio.01331-23 (PMC10746243; doi:10.1128/mbio.01331-23)
Supplement: Supplemental Material — Tables S1-S6; Fig. S1-S3. [file mbio.01331-23-s0001.pdf]

Supplementary Materials

Microbe-derived uremic solutes enhance thrombosis potential in the host

Ina Nemet,[1,2]† Masanori Funabashi,[3,4,5]†‡ Xinmin S. Li,[1,2] Mohammed Dwidar,[1,2] Naseer Sangwan,[1,2] Sarah M. Skye,[1,2]§ Kymberleigh A. Romano,[1,2]|| Tomas Cajka,[6]¶ Brittany D. Needham[7]#,** Sarkis K. Mazmanian[7], Adeline M. Hajjar[1,2], Federico E. Rey,[8] Oliver Fiehn,[6] W. H. Wilson Tang,[1,2,9] Michael A. Fischbach[3,4,5,10]* and Stanley L. Hazen,[1,2,9]*

[1]Department of Cardiovascular & Metabolic Sciences, Lerner Research Institute

[2]Center for Microbiome & Human Health, Cleveland Clinic, Cleveland, OH 44106, USA

[3]Department of Bioengineering, Stanford University, Stanford, CA 94305, USA

[4]Department of Microbiology and Immunology, Stanford University School of Medicine, Stanford University, Stanford, CA 94305, USA

[5]ChEM-H Institute, Stanford University, Stanford, CA 94305, USA

[6]West Coast Metabolomics Center, University of California, Davis, CA 95616, USA

[7]Departments of Biology and Biological Engineering, California Institute of Technology, Pasadena, CA, 91125, USA

[8]Department of Bacteriology, University of Wisconsin-Madison, Madison, WI 53706, USA

[9]Heart and Vascular Institute, Cleveland Clinic, Cleveland, OH 44106, USA

[10]Chan Zuckerberg Biohub, San Francisco, CA 94158, USA

†These authors contributed equally to this study

‡Current address: Translational Research Department, Daiichi Sankyo RD Novare Co., Ltd., Tokyo, 134-8630, Japan

§Current address: PwC, Mountain View, California, USA

||Current address: ProEd Communications, Cleveland, OH

¶Current address: Institute of Physiology of the Czech Academy of Sciences, Prague, 14200, Czech Republic

#Current address: Stark Neurosciences Research Institute, Indiana University School of Medicine, Indianapolis, IN 46202, USA

**Current address: Department of Anatomy, Cell Biology, and Physiology, Indiana University School of Medicine, Indianapolis, IN 46202, USA

*Correspondence: hazens@ccf.org and fischbach@fischbachgroup.org

Table S1. Baseline characteristics of the patients in the Discovery Cohort stratified by all-cause mortality (5 year)

Characteristics	Discovery cohort (n=1,149)	No death 5 years (n=1,023)	Yes death 5 years (n=126)	P value
Age (years)	64.03 ± 10.89	63.5 ± 10.7	67.8 ± 11.8	**<0.001**
Sex, male (%)	63.4	63.4	63.5	1
DM (%)	21.9	20.3	34.9	**<0.001**
History of hypertension (%)	71.8	70.2	84.9	**0.001**
History of hyperlipidemia (%)	85.4	86.2	78.6	**0.031**
Current smoking (%)	13.7	14.4	8.0	0.068
CAD (%)	75.5	74.8	81.0	0.159
Systolic blood pressure (mmHg)	132.0 (119.0-146.0)	133.0 (119.0-146.0)	130.5 (117.0-148.0)	0.833
HDL cholesterol (mg/dL)	34.4 (28.5-41.2)	34.5 (28.7-41.4)	32.7 (26.9-40.1)	0.064
LDL cholesterol (mg/dL)	96.0 (80.0-116.0)	96.0 (80.0-116.5)	95.0 (77.0-113.8)	0.231
TG (mg/dL)	122.0 (84.0-171.0)	122.0 (84.0-171.0)	117.0 (81.5-169.0)	0.814
C-reactive protein (mg/L)	2.31 (0.98-5.40)	2.14 (0.94-5.11)	3.92 (1.99-12.37)	**<0.001**
eGFR (ml/min/1.73 m^2)	89.64 (75.61-98.99)	90.85 (77.44-99.67)	79.31 (56.12-93.07)	**<0.001**
p-cresol (r.a.)	1663 (1240-2517)	1637 (1226-2425)	1931 (1389-3862)	**<0.001**
**Baseline medications**				
Aspirin (%)	76.7	77.1	73.0	0.359
ACE inhibitors (%)	49.9	48.3	62.7	**0.003**
Beta blocker (%)	65.3	64.1	74.6	**0.026**
Statin (%)	61.2	61.8	56.3	0.279

Discovery cohort consists of sequential stable subjects who underwent elective diagnostic coronary angiography (cardiac catheterization or coronary computed tomography) for evaluation of coronary artery disease (CAD). Continuous data are presented as mean ± standard deviation or median (interquartile range), categorical variables are presented as %; DM = Diabetes mellitus; HDL= high density lipoprotein; LDL = low density lipoprotein; TG = tryglycerides; eGFR = estimated glomerular filtration rate; r.a. = relative amount; ACE = angiotensin-converting-enzyme.

Table S2. Baseline characteristics of patients in the Validation Cohort stratified by all-cause mortality (5 year)

Characteristics	Validation Cohort (n=3,954)	No death 5 years (n=3,439)	Yes death 5 years (n=515)	P value
Age (years)	62.99 ± 10.91	62.11 ± 10.64	68.87 ± 10.85	**<0.001**
Sex male (%)	64.4	64.7	62.7	0.416
DM (%)	31.5	29.3	46.2	**<0.001**
History of hypertension (%)	71.0	69.9	78.4	**<0.001**
History of hyperlipidemia (%)	84.1	84.6	81.4	0.074
Current smoking (%)	12.8	13.0	11.3	0.312
CAD (%)	77.7	76.3	87.2	**<0.001**
Systolic blood pressure (mmHg)	133.0 (120.0-147.0)	133.0 (120.0-146.0)	134.0 (119.0-149.5)	0.376
HDL cholesterol (mg/dL)	34.2 (28.3-41.2)	34.5 (28.6-41.4)	32.2 (26.5-40.2)	**<0.001**
LDL cholesterol (mg/dL)	96.0 (78.0-117.0)	96.0 (78.0-117.5)	92.0 (74.0-113.0)	**0.001**
TG (mg/dL)	118.0 (85.0-169.0)	119.0 (85.0-169.0)	117.0 (86.5-172.5)	0.751
C-reactive protein (mg/L)	2.42 (1.04-5.91)	2.25 (0.97-5.35)	4.22 (1.96-10.63)	**<0.001**
eGFR (ml/min/1.73 m^2)	90.85 (75.77-100.22)	92.12 (78.70-101.10)	76.72 (55.20-91.81)	**<0.001**
IS (µM)	3.32 (2.04-5.19)	3.22 (1.98-4.98)	4.33 (2.64-7.22)	**<0.001**
*p*CS (µM)	18.95 (10.67-31.33)	18.18 (10.34-29.64)	25.91 (14.59-47.16)	**<0.001**
**Baseline medications**				
Aspirin (%)	73.8	74.7	67.6	**0.001**
ACE inhibitors (%)	50.1	48.8	59.0	**<0.001**
Beta blocker (%)	63.1	62.6	66.4	0.103
Statin (%)	60.1	60.5	57.9	0.284

Validation Cohort was a sequential series of non-overlapping stable subjects undergoing elective diagnostic cardiac evaluations for coronary artery disease (CAD) risk assessment. Continuous data are presented as mean ± standard deviation or median (interquartile range), categorical variables are presented as %; DM = diabetes mellitus; HDL= high density lipoprotein; LDL = low density lipoprotein; TG = tryglycerides; IS=indoxyl sulfate; *p*CS=*p*-cresol sulfate eGFR = estimated glomerular filtration rate; ACE = angiotensin-converting-enzyme.

Table S3. Hazard ratios (HR) for 5-year all-cause mortality based on the Cox proportional hazards regression analysis (Sensitivity analysis)

***p*-Cresol sulfate**<br>**P-interaction=0.88**	*p*-cresol sulfate quartiles (μM)			
eGFR≥60 ml/min/1.73 m^2	Q_1<br>(0.01-10.11)	Q_2<br>(10.12-17.74)	Q_3<br>(17.75-28.29)	Q_4<br>(28.30-146.79)
HR (95% CI)	1	1.04(0.75-1.45)	1.37(1.01-1.86)*	1.85(1.38-2.48)***
eGFR<60 ml/min/1.73 m^2	Q_1<br>(0.21-25.91)	Q_2<br>(25.92-44.89)	Q_3<br>(44.90-69.26)	Q_4<br>(69.27-375.23)
HR (95% CI)	1	1.00(0.59-1.69)	1.46(0.90-2.38)	2.07(1.32-3.25)**
**Indoxyl sulfate**<br>**P-interaction=0.48**	indoxyl sulfate quartiles (μM)			
eGFR≥60 ml/min/1.73 m^2	Q_1<br>(0.001-1.95)	Q_2<br>(1.96-3.12)	Q_3<br>(3.13-4.72)	Q_4<br>(4.73-40.34)
HR (95% CI)	1	1.31(0.95-1.80)	1.42(1.04-1.94)*	1.68(1.24-2.28)***
eGFR<60 ml/min/1.73 m^2	Q_1<br>(0.001-4.24)	Q_2<br>(4.25-7.07)	Q_3<br>(7.08-12.20)	Q_4<br>(12.21-233.95)
HR (95% CI)	1	1.44(0.85-2.46)	1.66(1.00-2.74)*	2.60(1.62-4.16)***

eGFR = estimated glomerular filtration rate; HR = hazard ratio; CI =confidence intervals; *P<0.05, **P<0.01, ***P<0.001

Table S4. Bacterial strains used in this study

Strain	Specific Genotype	Plasmid for integration	Source
B. thetaiotaomicron VPI-5482 Δ*tdk*			Sonnenburg Lab, Stanford University
B. vulgatus ATCC 8482			ATCC
C. sporogenes ATCC 15579			ATCC
Clostridium sp. D5			BEI
Clostridioides difficile JIR8094			Shen Lab, Tufts University
Blautia hydrogenotrophica DSM 10507			DSMZ
E. coli S17-1 λ pir			Sonnenburg Lab, Stanford University
B. thetaiotaomicron VPI-5482 Δ*tdk*	Δ*BT0331*		Zhu et al. 2022
B. thetaiotaomicron VPI-5482 Δ*tdk*	Δ*BT0430*		Zhu et al. 2022
B. thetaiotaomicron VPI-5482 Δ*tdk*	Δ*BT2836*		Zhu et al. 2022
B. thetaiotaomicron VPI-5482 Δ*tdk*	Δ*BT0331* Δ*BT0431*		Zhu et al. 2022
B. thetaiotaomicron VPI-5482 Δ*tdk*	Δ*BT0431* Δ*BT2836*		Zhu et al. 2022
B. thetaiotaomicron VPI-5482 Δ*tdk*	Δ*BT0331* Δ*BT0430* Δ*BT2836*		Zhu et al. 2022
B. thetaiotaomicron VPI-5482 Δ*tdk*	Δ*BT0430* *BT0430* (complementation)	pMFT01	this study
B. thetaiotaomicron VPI-5482 Δ*tdk*	Δ*BT1492*		lab stock
B. thetaiotaomicron VPI-5482 Δ*tdk*	Δ*BT1492* *HpdBCA*	pMFT02	this study
B. thetaiotaomicron VPI-5482 Δ*tdk*	*BT1492* *BT1492* (additional two copies)	pMFT03 & pMFT04	this study
B. thetaiotaomicron VPI-5482 Δ*tdk*	*BT1492* *HpdBCA*	pMFT02 & pMFT04	this study

Table S5. List of plasmids used in this study

Plasmid	Backbone	Promoter	Inserted gene	Source
pNBU2_bla_tetQb				Sonnenburg Lab, Stanford University
pMFT01	pNBU2_bla_ermGb	σ70 promoter	*BT0430*	this study
pMFT02	pNBU2_bla_ermGb	phage promoter	*HpdBCA*	this study
pMFT03	pNBU2_bla_ermGb	phage promoter	*BT1492*	this study
pMFT04	pNBU2_bla_tetQb	phage promoter	*BT1492*	this study

Table S6. List of primers used in this study

Primer #	Primer Name	Sequence (5'-3')	Restriction Site
1	BT0430_comp_F	GCGTCTAGAATGAGCAAGCAACTCTTACTTG	*Xba*I
2	BT0430_comp_R	GCGGGATCCTTACTTACTTCTTTTTTTGCGTGC	*Bam*HI
4	pNBU2_F	TCTAGAACTAGTGGATCCCC	
5	pNBU2_R	GCGGCCGCCACCGCGG	
6	3'pNBU2_phageP_F	CCTCCACCGCGGTGGCGGCCGCGATAAAACGAAAGGCTCAG	
7	phageP_5'HpdBCA_R	ACTTTGACTCATCTACTTTGTTTCTTTCGACAC	
8	3'phageP_HpdBCA_F	GAAACAAAGTAGATGAGTCAAAGTAAAGAAGAC	
9	5'pNBU2_HpdBCA_R	CGGGGGATCCACTAGTTCTAGATTAGAAAGCTGTCTCATGAC	
10	phageP_5'BT1492_R	AGGTAATTCCATCTACTTTGTTTCTTTCGACAC	
11	3'phageP_BT1492_F	GAAACAAAGTAGATGGAATTACCTTTTGCTGAATC	
12	5'pNBU2_BT1492_R	CGGGGGATCCACTAGTTCTAGACTATACAGACAATCGTTCTAA	

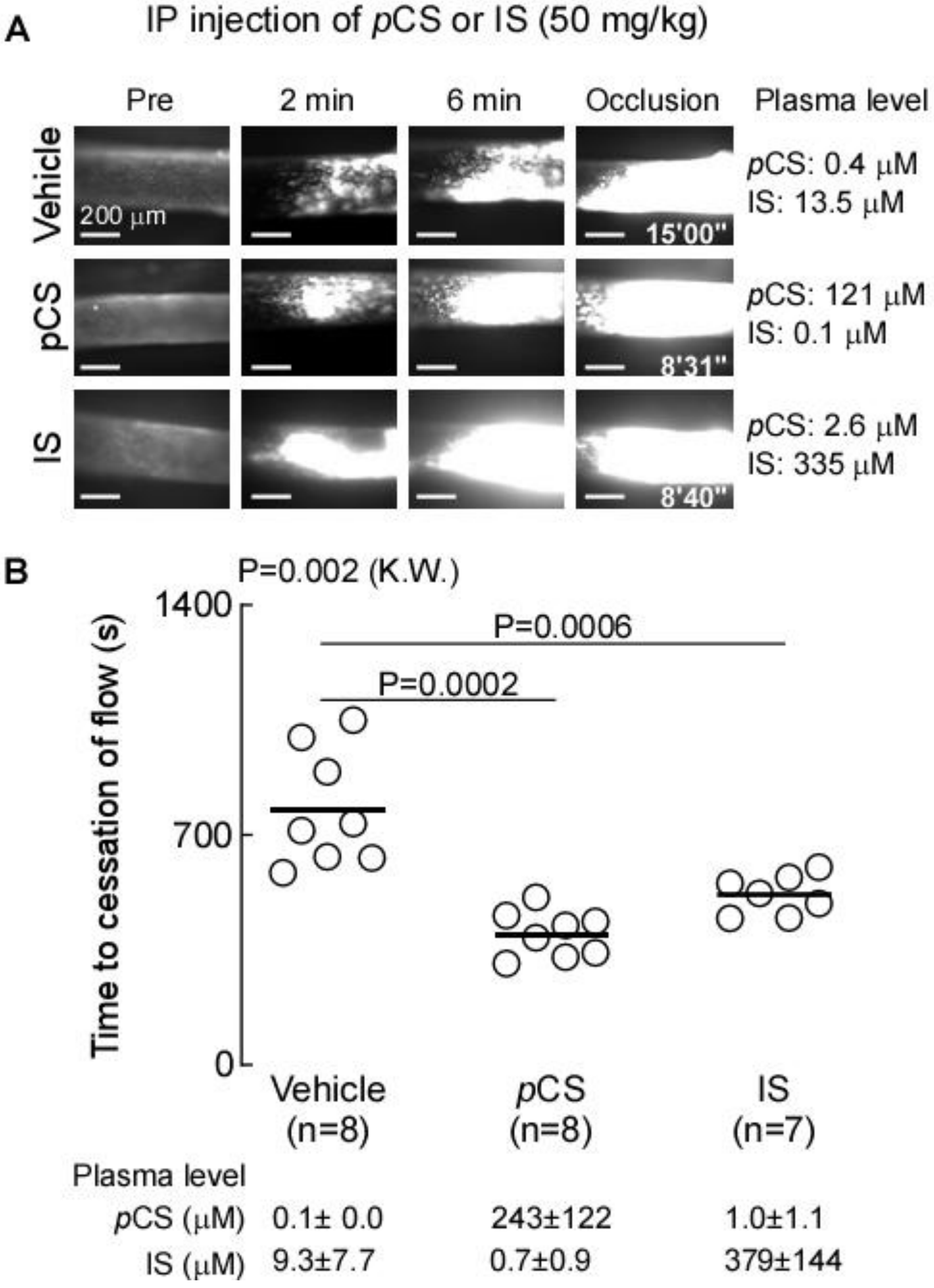

Fig. S1. ***p*-Cresol sulfate (*p*CS) and indoxyl sulfate (IS) are associated with increased *in vivo* thrombosis potential**. (**A,B**) *In vivo* thrombosis potential was measured by the $FeCl_3$-induced carotid artery injury model in response to *p*-cresol sulfate or indoxyl sulfate (versus normal saline) injected *i.p.* into mice (50 mg/kg). Shown are representative (**A**) vital microscopy images of carotid artery thrombus formation at the indicated time points following arterial injury, and (**B**) time to cessation of blood flow in mice from the indicated groups. Bar represents mean time to cessation of blood flow within the indicated group. Plasma levels of *p*CS and IS at time of thrombosis model are indicated. Significance was measured by Kruskal-Wallis test.

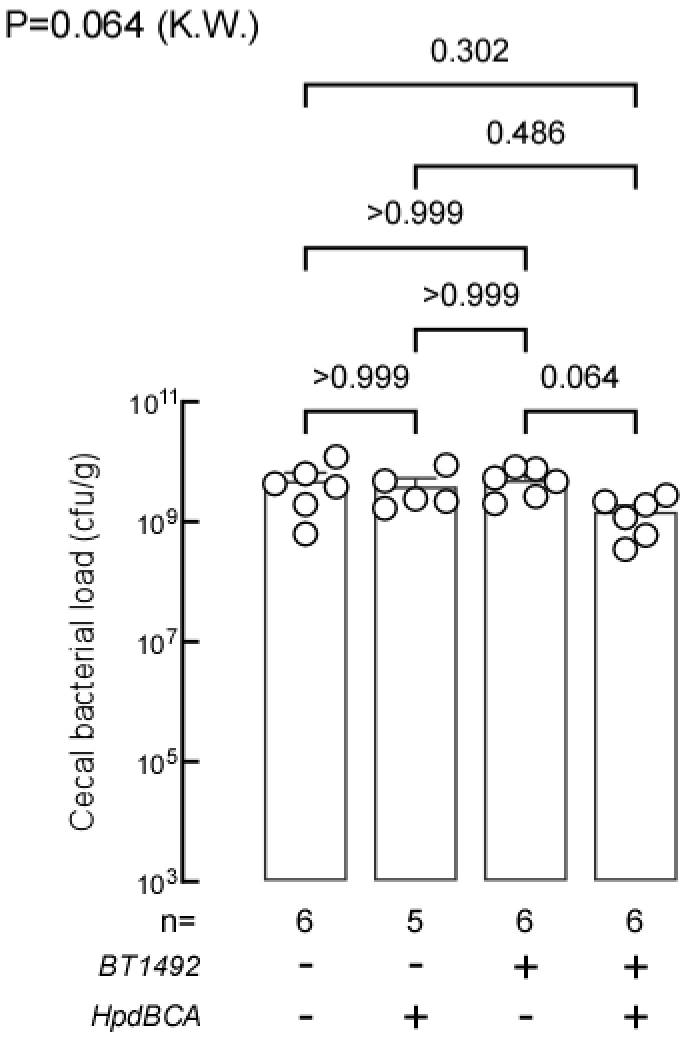

Fig. S2. ***B. thetaiotaomicron*** **strains with different capacities for *p*-cresol and indole production did not cause significant change in their degree of colonization in gnotobiotic mice.** Germ-free mice were randomized and colonized with *B. thetaiotaomicron* mutants as indicated. Ceca were weighed, thawed under anaerobic conditions, and suspended in PBS buffer. Dilutions of the cecal suspensions were then plated on BHI agar plates, incubated for 72 h at 37 °C under anaerobic conditions for colony counting. “n” represents the number of mice in each group. Bars represent mean±SE. Significance was measured with a Kruskal-Wallis (K.W.) test followed with Dunn's multiple comparisons test.

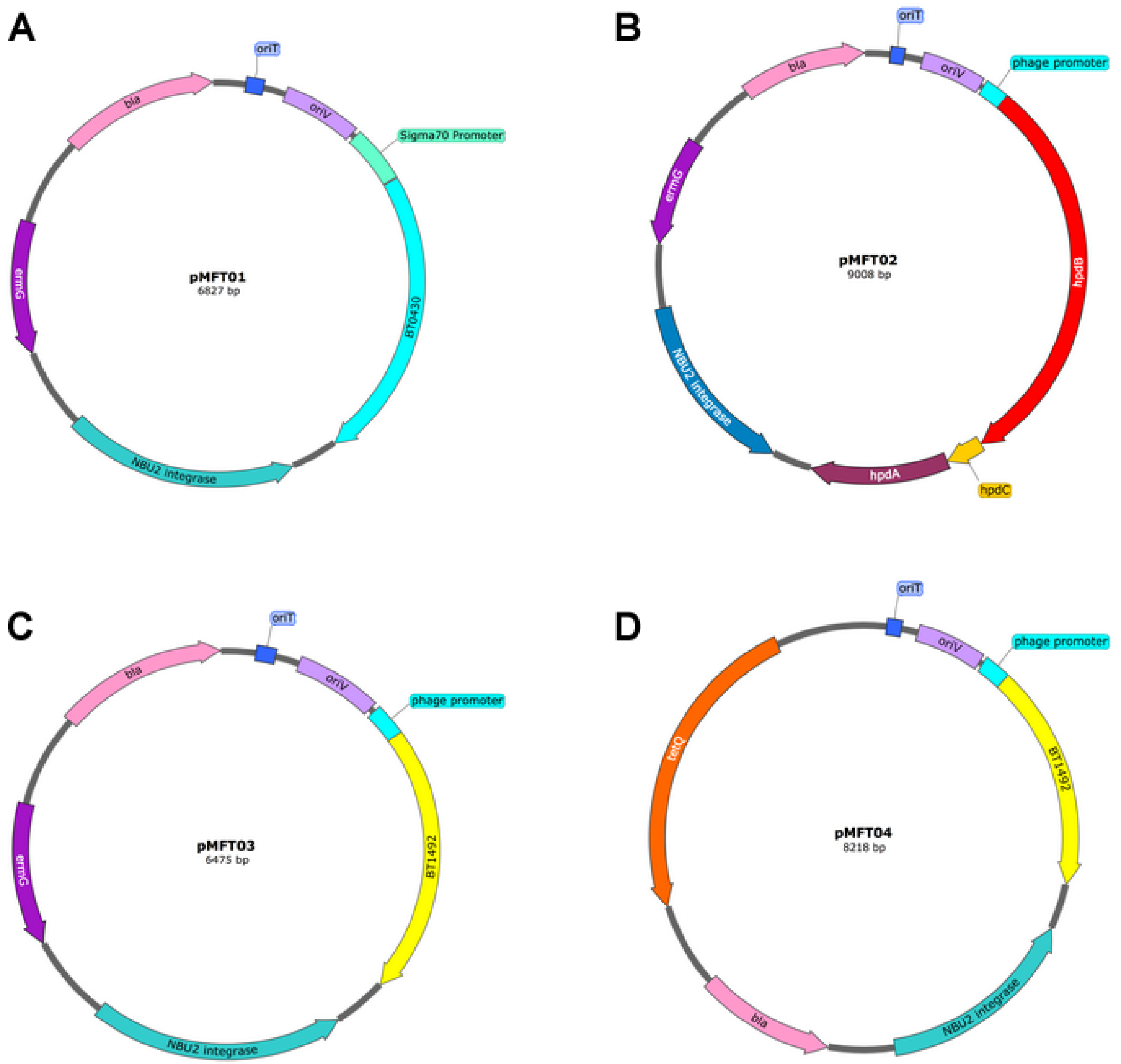

Fig. S3. Maps for integration vectors. (**A**) BT0430 was cloned into pNBU2 vector with erythromycin resistant marker under σ70 promoter. (**B**) *hpdBCA* operon was cloned into pNBU2 vector with erythromycin resistant marker under the phage promoter. (**C**) *BT1492* gene was cloned into pNBU2 vector with erythromycin (**C**) tetracycline (**D**) resistant marker under the phage promoter.
